# Loneliness as a Key Factor in Depressive and Anxiety Symptoms among Vietnamese Migrants in Japan during the COVID-19 Pandemic: A Cross-sectional Study

**DOI:** 10.31662/jmaj.2024-0134

**Published:** 2025-03-07

**Authors:** Tadashi Yamashita, Pham Nguyen Quy, Emi Nogami, Chika Yamada, Kuniyasu Kamiya, Kenji Kato

**Affiliations:** 1 Faculty of Nursing, Kobe City College of Nursing, Kobe, Japan; 2 Department of Medical Oncology, Kyoto Miniren Central Hospital, Kyoto, Japan; 3 Department of Social Welfare, School of Psychology and Social Welfare, Mukogawa Women’s University, Nishinomiya, Japan; 4 Department of Environmental Coexistence, Center for Southeast Asian Studies, Kyoto University, Kyoto, Japan; 5 Faculty of Nursing, Kobe Women’s University, Kobe, Japan

**Keywords:** depression, anxiety disorders, loneliness, Vietnamese migrants, Japan

## Abstract

**Introduction:**

Loneliness is a major factor hindering the health of migrants. There is concern that social changes due to the COVID-19 pandemic, in addition to the acculturation gap with their host country, exacerbated loneliness among Vietnamese migrants in Japan. Therefore, this study aimed to clarify the prevalence of loneliness and to evaluate the relationship with depressive and anxiety symptoms among Vietnamese migrants in Japan.

**Methods:**

We used a cross-sectional study design with a self-administered questionnaire. The data were collected from May 2 to June 6, 2022. The target population for this study was Vietnamese migrants living in Japan, 213 of whom were included in the analysis. The questionnaire consisted of items regarding participants’ characteristics, socioeconomic status, social support, Patient Health Questionnaire-9 scores, Generalized Anxiety Disorder-7 scores, and University of California Los Angeles 3-Item Loneliness Scale scores. Logistic regression analysis was performed with depressive and anxiety symptoms as dependent variables and loneliness and other socioeconomic factors as independent variables.

**Results:**

The mean age of the participants was 26.8 ± 4.4 years. The study included 112 men (52.6%) and 101 women (47.4%). Their mean years of residence in Japan was 4.4 ± 2.5 years. The mean score on the University of California Los Angeles 3-Item Loneliness Scale was 7.2 ± 2.4. Multivariate logistic regression analysis revealed that depressive symptoms were associated with loneliness (odds ratio [OR]: 1.797; 95% confidence interval [CI]: 1.434-2.251). Similarly, factors associated with anxiety disorders included loneliness (OR: 2.051; 95% CI: 0.204-1.750).

**Conclusions:**

Loneliness is a significant factor contributing to depressive and anxiety symptoms among Vietnamese migrants in Japan. Therefore, reducing loneliness is essential to improving the mental health and overall well-being of the rapidly growing Vietnamese migrant population.

## Introduction

Migrants are vulnerable to various aspects of society, including their socioeconomic background, social status, language ability, and access to healthcare ^(1), (2)^. These vulnerabilities were exacerbated during the COVID-19 pandemic, which disproportionately affected migrants’ mental health worldwide due to increased social isolation, job insecurity, and limited access to support services ^(3), (4), (5)^.

In Japan, Vietnamese migrants stand out due to the rapid increase in their numbers and their unique challenges. As of June 2023, there were 520,154 Vietnamese migrants in Japan, a sevenfold increase compared with 2013, making them the second largest foreign population after Chinese nationals ^(6)^. Many Vietnamese migrants come to Japan under the Technical Intern Training Program, which often leaves them in precarious economic conditions due to the recruitment debts averaging 700,000 yen and limited access to social support systems ^(7)^. These vulnerabilities were further magnified during the COVID-19 pandemic, with widespread layoffs and economic downturns disproportionately affecting Vietnamese migrants. This economic instability, coupled with cultural barriers and limited access to healthcare, has significantly contributed to mental health challenges within this population ^(8)^.

Our research group has been conducting a prospective observational study on Vietnamese migrants in Japan since 2021, reporting on changes in their socioeconomic conditions and mental health status during the COVID-19 pandemic ^(9)^. This study builds on the findings of our cohort research and represents the first analysis specifically focusing on loneliness among Vietnamese migrants in Japan. Using data from the cohort study, this research provides a comprehensive understanding of the mental health challenges faced by this population, with particular attention to loneliness.

Loneliness is a critical determinant of health among migrants and has been found to increase the risk of mental health disorders, such as depression, anxiety, alcoholism, and sleep disorders ^(10), (11), (12)^. For Vietnamese migrants in Japan, loneliness is exacerbated by cultural misunderstandings, limited opportunities for meaningful social interactions, and systemic discrimination ^(13), (14)^. The relationship between loneliness and mental health issues, such as depressive and anxiety symptoms, is well-documented, and loneliness is particularly significant for this population due to their unique sociocultural and economic circumstances. Furthermore, addressing loneliness may offer a strategic entry point for mental health interventions aimed at improving the well-being of Vietnamese migrants.

Understanding the effect of the pandemic on Vietnamese migrants requires contextualizing their experiences within prepandemic and pandemic-specific vulnerabilities. Before the pandemic, loneliness and social isolation among migrants were already recognized as significant contributors to mental health issues, including depression and anxiety ^(15), (16)^. Subsequently, the pandemic magnified these issues by imposing additional social restrictions and exacerbating economic stress. Comparative studies indicate that migrants’ mental health deteriorated more severely during the pandemic compared with non-pandemic periods, highlighting the role of the pandemic as a stress amplifier ^(17), (18)^. This is particularly evident among Vietnamese migrants in Japan who faced compounded challenges due to their reliance on low-wage jobs, language barriers, and limited access to culturally sensitive healthcare services. Understanding how the pandemic uniquely affected this population provides critical insights into their vulnerabilities and informs strategies for their post-pandemic recovery.

Given these challenges, this study aims to investigate the extent of loneliness among Vietnamese migrants in Japan and its role as a key factor in exacerbating depression and anxiety symptoms. Clarifying the current state of loneliness and its influence on depression and anxiety symptoms, this research provides critical evidence for developing culturally sensitive and effective mental health support strategies for this growing population.

## Materials and Methods

### Study design

**Figure 1.**
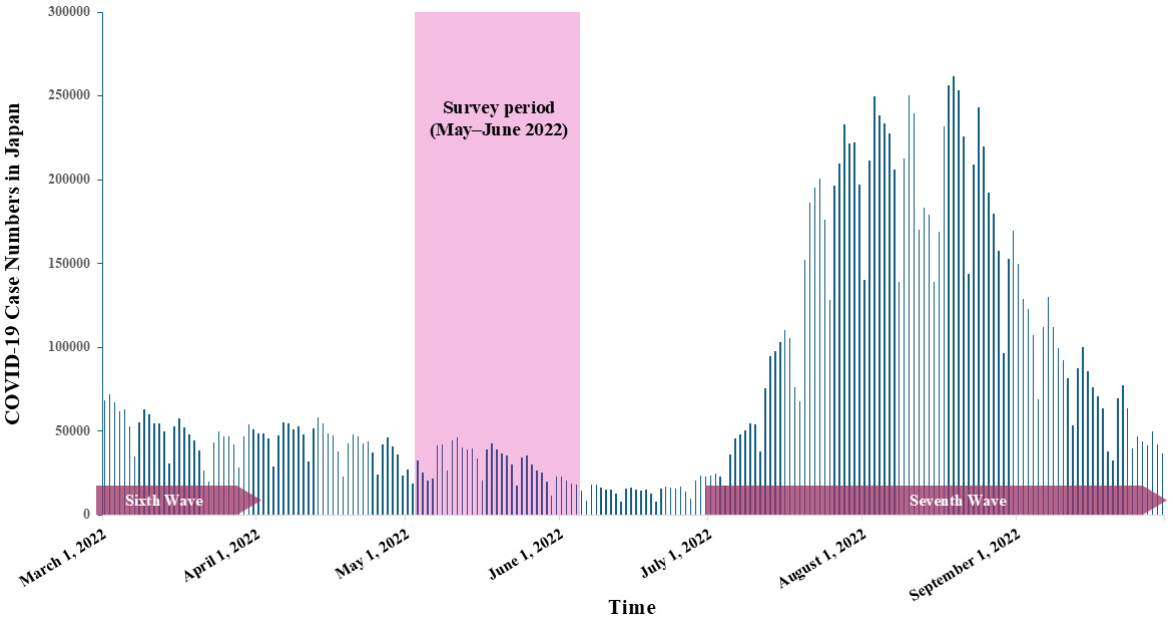
Trends in COVID-19 case numbers, pandemic waves in Japan, and survey period. COVID-19: coronavirus disease 2019.

This study used a cross-sectional research design with a self-administered questionnaire made accessible online through Survey Monkey (Momentive Inc., San Mateo, CA, USA). Questionnaires translated into Vietnamese were disseminated through social networking communities, specifically on Facebook (Meta-Platforms Inc.), which are frequently visited by Vietnamese migrants residing in Japan. A comprehensive overview of the study, along with ethical considerations for the participants, was communicated through a promotional leaflet posted on the relevant Facebook pages. The data collection period occurred from May 2 to June 6, 2022, during the interval between the sixth and seventh waves of the COVID-19 pandemic in Japan (Figure 1). Since 2021, our research group has been conducting a prospective observational study on Vietnamese migrants in Japan, focusing on changes in their socioeconomic conditions and mental health status during the COVID-19 pandemic. This study builds on our ongoing cohort research and specifically uses data from the 2022 survey to analyze loneliness among Vietnamese migrants for the first time within this cohort. Evaluating the role of loneliness, this study provides new insights into its impact as a key factor in exacerbating depression and anxiety symptoms among this population. This study was approved by the Kobe City College of Nursing Research Ethics Review Committee (approval number: 20124-05).

### Participants

The study participants were Vietnamese migrants living in Japan. Participation criteria were Vietnamese aged ≥18 years and living in Japan with a resident status. Exclusion criteria were those who had “temporary visa” status and those who could not answer the questionnaire by themselves.

The sample size required for this study was calculated using G*Power version 3.1. With an odds ratio (OR) of 2.5, a significance level of 0.05, and a power of 0.80 set by G*Power, the sample size was determined to be 200. An OR of 2.5 was selected to detect a moderate-to-strong association, which is often used in similar studies investigating health-related outcomes ^(19)^. Considering the missing response rate of 60% for online questionnaires ^(20)^, the minimum sample size for collection was determined to be 320 (200+200×0.6) participants. A forced-response design, which prevents participants from proceeding without completing all items, was not implemented in this study. This decision was made to prioritize participant comfort and minimize the risk of survey abandonment, as forced-response designs can increase respondent fatigue, particularly in surveys addressing sensitive topics, such as mental health. To address missing data, a complete-case analysis approach was used. This method was chosen to ensure that all included data were fully observed, thereby reducing potential inaccuracies introduced by imputation methods and maintaining the clarity and interpretability of the results.

Missing data were addressed through predefined exclusion criteria, which removed responses with incomplete data for key variables, including participant characteristics, socioeconomic status, Patient Health Questionnaire-9 (PHQ-9) scores, Generalized Anxiety Disorder-7 (GAD-7) scores, and University of California Los Angeles 3-Item Loneliness Scale (UCLA 3-ILS) scores. Therefore, after excluding incomplete cases, 213 of 287 collected responses were included in the analysis.

### Questionnaire

The questionnaire consisted of participants’ characteristics (age, sex, duration of residence in Japan, highest education level, Japanese language proficiency, and status of residence), subjective participants’ socioeconomic status, social support (existence of someone with whom the participant could discuss their health), PHQ-9 scores, GAD-7 scores, and the UCLA 3-ILS scores.

Clinical depression was assessed with the PHQ-9 and anxiety symptoms with the GAD-7. Both scales used a 4-point Likert format (0-3), yielding total scores of 0 to 27 for the PHQ-9 and 0 to 21 for the GAD-7. Scores of more than or equal to 10 on either scale indicated clinically significant conditions ^(21), (22)^. Previous studies concluded that the PHQ-9 and GAD-7 are valid and reliable instruments for assessing depression in the Vietnamese population ^(23), (24)^. The UCLA 3-ILS has been widely used to measure loneliness in a simplified form, using a 4-point scale (1 = never, 2 = rarely, 3 = sometimes, and 4 = always). Scores range from 3 to 12, with higher scores indicating greater loneliness severity ^(25)^. The instrument was translated and back-translated into Vietnamese by separate groups of bilinguals working in the field of psychology. Cronbach’s alpha coefficient was used to evaluate reliability in this study, and the value obtained for the UCLA 3-ILS was 0.91. The questionnaire was administered entirely in Vietnamese.

### Analyses

Descriptive statistics were used to analyze each value. Subsequently, a single regression analysis with depressive (PHQ-9 ≥ 10 points) and anxiety symptoms (GAD-7 ≥ 10 points) as dependent variables and loneliness (UCLA 3-ILS score range, 3-12) as the independent variable followed by a logistic regression analysis was conducted. To assess the potential impact of multicollinearity among the independent variables, we calculated the variance inflation factor for each explanatory variable. All variance inflation factor values were below 10, indicating that multicollinearity was not a concern in this study. The independent variables were age, sex, length of stay in Japan, highest education level (three groups: college or higher, vocational school, and high school or lower), residence status (two groups: international students and non-students), Japanese language proficiency (two groups: higher proficiency and lower proficiency), subjective economic status (two groups: strict and good), and having someone with whom to discuss one’s health (two groups: yes and no). The forced entry method was used for variable selection in the logistic regression model. Independent variables were selected based on unique characteristics of immigrants residing in Japan, such as duration of residence and Japanese language proficiency. In addition, previous studies provided a rationale for including specific variables in the model. For instance, Yamashita et al. ^(13)^ identified associations between depressive and anxiety symptoms among Vietnamese residents in Japan and factors such as subjective socioeconomic status and the presence of someone with whom they could discuss health concerns. Similarly, Uezato et al. ^(14)^ highlighted a relationship between mental distress and residency status among Vietnamese immigrants. Building on their findings, this study incorporated subjective socioeconomic status, the existence of someone with whom to discuss health concerns, and residency status as independent variables in the regression analysis. Bootstrapping was used to account for overfitting the logistic regression model. The significance level was set at *P* less than 0.01. All statistical analyses were performed using the SPSS software (version 26.0; IBM Corp., Armonk, NY, USA).

## Results

As shown in Table 1, among all participants (n = 213), the mean age was 26.8 ± 4.4 years, and 52.6% were men. The mean duration of residence in Japan was 4.4 ± 2.5 years. Regarding marital status, 24.9% were married, and 75.1% were single, divorced, or widowed. The most common visa types were residence based on employment (34.7%) and international students (30.0%). Most participants had a college or university education (41.8%), and 62.4% were classified as having higher Japanese language proficiency. In addition, 43.2% reported socioeconomic difficulties, and 54.0% lacked someone with whom they could discuss their health. The mean UCLA 3-ILS score was 7.2 ± 2.4. Overall, 24.9% had PHQ-9 scores ≥10, and 14.1% had GAD-7 scores ≥10. Participants with PHQ-9 scores ≥10 (n = 53) had a mean age of 26.0 ± 4.1 years, and 54.7% were men. The mean duration of residence was 3.7 ± 2.1 years, and 13.2% were married. The most common visa types were international students (35.8%) and employment-based visa holders (34.0%). Socioeconomic difficulties were reported by 62.3%, and 73.6% lacked someone with whom they could discuss their health. The mean UCLA 3-ILS score was 9.2 ± 1.7. Participants with GAD-7 scores ≥10 (n = 30) had a mean age of 27.1 ± 4.4 years, and 63.3% were men. The mean duration of residence was 3.5 ± 1.5 years, and 6.7% were married. The most common visa types were international students (50.0%) and employment-based visa holders (20.0%). Socioeconomic difficulties were reported by 73.3%, and 60.0% lacked someone with whom they could discuss their health. The mean UCLA 3-ILS score was 9.5 ± 1.8 (Table 1).

**Table 1.** Characteristics and Depression, Anxiety, and Loneliness Status among Vietnamese Migrants in Japan.

Items		All (N = 213)		PHQ-9 ≧ 10 points (n = 53)		GAD-7 ≧ 10 points (n = 30)	
		n	%	n	%	n	%
Age (year)	(Mean ± SD)	26.8 ± 4.4		26.0 ± 4.1		27.1 ± 4.4	
Sex	Male	112	52.6	29	54.7	19	63.3
	Female	101	47.4	24	45.3	11	36.7
Duration of residence in Japan (year)	(Mean ± SD)	4.4 ± 2.5		3.7 ± 2.1		3.5 ± 1.5	
Marital status	Married	53	24.9	7	13.2	2	6.7
	Single, divorced, or widowed	160	75.1	46	86.8	28	93.3
Visa status	Residence based on the nature of activities	24	11.3	6	11.3	4	13.3
	Residence based on employment	74	34.7	18	34.0	6	20.0
	International student	64	30.0	19	35.8	15	50.0
	Technical intern Training (*Ginojisshu*)	25	11.7	4	7.5	2	6.7
	Skilled labor (*Tokuteiginou*)	20	9.4	5	9.4	3	10.0
	Other	6	2.8	1	1.9	0	0.0
Japanese language proficiency	Higher proficiency	133	62.4	30	56.6	18	60.0
	Lower proficiency	80	37.6	23	43.4	12	40.0
Education level	Junior high school and under	42	19.7	12	22.6	7	23.3
	High school	60	28.2	17	32.1	8	26.7
	Technical school	1	0.5	0	0.0	0	0.0
	College (2 years) or university	89	41.8	21	39.6	13	43.3
	Graduate school	21	9.9	3	5.7	2	6.7
Subjective socioeconomic status	Good/general	121	56.8	20	37.7	8	26.7
	Difficult	92	43.2	33	62.3	22	73.3
Existence of someone to discuss their health with	No	115	54.0	39	73.6	18	60.0
PHQ-9 (0-27 points)	0-9 points	160	75.1	N/A	N/A	6	20.0
	10-27 points	53	24.9	N/A	N/A	24	80.0
GAD-7 (0-21 points)	0-9 points	183	85.9	29	54.7	N/A	N/A
	10-21 points	30	14.1	24	45.3	N/A	N/A
UCLA 3-ILS (3-12 points)	(Mean ± SD)	7.2 ± 2.4		9.2 ± 1.7		9.5 ± 1.8	

GAD-7: Generalized Anxiety Disorder-7; N/A: not available; PHQ-9: Patient Health Questionnaire-9; UCLA 3-ILS: University of California Los Angeles 3-Item Loneliness Scale.

Table 2 shows the results of the unadjusted logistic regression analysis. Depressive symptoms were significantly associated with subjective socioeconomic status (OR: 2.825; 95% confidence interval [CI]: 1.487-5.365; *P* = 0.002) and having no one with whom to discuss health-related issues (OR: 3.079; 95% CI: 1.552-6.108; *P* = 0.001). In addition, loneliness measured by the UCLA 3-ILS had an association with depressive symptoms (OR: 1.892; 95% CI: 1.531-2.338; *P* < 0.001). Moreover, subjective socioeconomic status (OR: 4.439; 95% CI: 1.874-10.516; *P* = 0.001) and loneliness (OR: 1.935; 95% CI: 1.482-2.526; *P* < 0.001) were significant predictors for anxiety disorders.

**Table 2.** Unadjusted Logistic Regression Analysis with Clinical Depression and Anxiety Disorders as Outcomes (N = 213).

Variables		Depression				Anxiety disorder			
		Odds ratio	[95% CI]		*P*	Odds ratio	[95% CI]		*P*
Age		0.943	0.875	1.017	0.130	0.866	0.776	0.966	0.010
Sex	Female	1.000			0.720	1.000			0.207
	Male	0.892	0.478	1.664		0.598	0.270	1.328	
Duration of residence in Japan		0.848	0.724	0.993	0.040	0.791	0.630	0.992	0.043
Education level	College, university, or higher	1.000				1.000			
	Technical school	1.433	0.697	2.947	0.328	0.985	0.392	2.476	0.974
	High school or lower	1.450	0.647	3.252	0.367	1.280	0.482	3.400	0.620
Japanese language proficiency	Higher proficiency	1.000			0.312	1.000			0.766
	Lower proficiency	1.385	0.736	2.607		1.127	0.512	2.483	
Subjective socioeconomic status	Good/general	1.000			0.002^a^	1.000			0.001^a^
	Difficult	2.825	1.487	5.365		4.439	1.874	10.516	
Existence of someone to discuss their health with	Yes	1.000			0.001^a^	1.000			0.477
	No	3.079	1.552	6.108		1.330	0.606	2.919	
Status of residence	International students	1.000			0.289	1.000			0.012
	Non-students	0.700	0.362	1.353		0.366	0.166	0.803	
UCLA 3-ILS (3-12 points)		1.892	1.531	2.338	0.000^a^	1.935	1.482	2.526	0.000^a^

CI: confidence interval; UCLA 3-ILS: University of California Los Angeles 3-Item Loneliness Scale. ^a^*P* < 0.01.

Table 3 presents the results of the multivariate logistic regression analysis. In this model, depressive symptoms were significantly associated with loneliness (OR: 1.797; 95% CI: 1.434-2.251; *P* < 0.001). Similarly, loneliness was strongly associated with anxiety disorders (OR: 2.051; 95% CI: 0.204-1.750; *P* < 0.001).

**Table 3.** Adjusted Logistic Regression Analysis with Clinical Depression and Anxiety Disorders as Outcomes (N = 213).

Variables		Depression				Anxiety disorder			
		Odds ratio	[95% CI]		*P*	Odds ratio	[95% CI]		*P*
Age		0.991	0.882	1.113	0.878	0.858	0.727	1.013	0.070
Sex	Female	1.00			0.911	1.00			0.119
	Male	1.044	0.491	2.222		0.463	0.176	1.219	
Duration of residence in Japan		0.880	0.711	1.088	0.238	0.646	0.695	1.253	0.646
Education level	College, university, or higher	1.00				1.00			
	Technical school	1.011	0.422	2.422	0.980	0.378	0.111	1.286	0.119
	High school or lower	0.991	0.342	2.875	0.987	0.278	0.067	1.147	0.077
Japanese language proficiency	Higher proficiency	1.00			0.555	1.00			0.889
	Lower proficiency	1.262	0.583	2.732		0.931	0.342	2.534	
Subjective socioeconomic status	Good/general	1.00			0.673	1.00			0.148
	Difficult	1.187	0.536	2.628		2.191	0.757	6.347	
Existence of someone to discuss their health with	Yes	1.00			0.156	1.00			0.124
	No	1.786	0.801	3.980		0.431	0.147	1.258	
Status of residence	International students	1.00			0.878	1.00			0.347
	Non-students	0.931	0.375	2.312		0.597	0.204	1.750	
UCLA 3-ILS (3-12 points)		1.797	1.434	2.251	0.000^a^	2.051	0.204	1.750	0.000^a^

Independent variables included age, sex, length of stay in Japan, highest education level, status of residence, Japanese language proficiency, subjective economic status, and having someone to discuss their health with. CI: confidence interval; UCLA 3-ILS: University of California Los Angeles 3-Item Loneliness Scale. ^a^*P* < 0.01.

## Discussion

The mental health problems of the rapidly growing Vietnamese migrant population in Japan are an extremely important public health issue. This study contributes to the understanding of loneliness among Vietnamese migrants in Japan, highlighting its role as a significant factor in exacerbating mental health issues. These findings underscore the need to prioritize loneliness as a key aspect when addressing the mental health and well-being of this population, particularly as targeted solutions remain underdeveloped.

Loneliness has been found to precipitate various mental disorders, including depression, alcoholism, and sleep disturbances ^(12)^. Among migrants, loneliness is a well-documented risk factor for depressive symptoms. Previous studies, such as those evaluating older migrants in the United States, have revealed that loneliness correlates strongly with depressive symptoms, particularly in individuals who migrated later in life ^(26)^. Globally, the COVID-19 pandemic has been associated with increased levels of loneliness ^(27)^. A longitudinal study in Switzerland reported a rise in emotional loneliness during the pandemic ^(28)^, and a cross-sectional study involving 20,398 respondents across 101 countries found that severe loneliness prevalence increased from 6% before the pandemic to 21% during the pandemic ^(29)^. These findings are echoed in studies focusing on the Japanese population, in which loneliness scores remained persistently high during and after the pandemic ^(30)^.

Despite these global trends, studies specifically evaluating loneliness among migrant populations during the pandemic remain limited. Research on African migrants in Australia highlighted the disruptive impact of lockdowns on community cohesion, leading to increased loneliness ^(31)^. However, few studies have explored the unique experiences of migrants in Japan, including Vietnamese migrants, during this period. This gap in the literature is significant, as Vietnamese migrants in Japan face distinct challenges, such as cultural and linguistic barriers, limited social networks, and restrictive residency conditions.

Vietnamese migrants in Japan are predominantly technical intern trainees or international students, with technical interns comprising the most common residency status ^(6)^. Their temporary residency―typically limited to 5 years―and unfamiliarity with Japanese culture hinder the formation of meaningful social relationships with their Japanese colleagues and employers, contributing to social isolation. In addition, cultural stigmas surrounding mental health further exacerbate their challenges. Mental disorders are often perceived as a sign of personal weakness among Vietnamese communities, as evidenced by studies on Vietnamese Americans ^(32)^. This stigma likely acts as a barrier preventing Vietnamese migrants in Japan from seeking psychiatric care, with only 1.4% of all migrant patients receiving such care in the country ^(33)^.

The unique vulnerabilities of Vietnamese migrants during the COVID-19 pandemic―including language barriers, social disconnection, and cultural isolation―may have amplified their experiences of loneliness compared with other groups. Although Japanese workers faced increased work-related stress, their cultural familiarity and language fluency likely offered some protection against loneliness. Similarly, other migrant groups, such as Chinese or Filipino migrants, may have benefited from well-established ethnic communities that provided social support. In contrast, Vietnamese migrants, lacking such resources, seem to have been disproportionately affected by loneliness.

Policy recommendations aimed at reducing loneliness and improving mental health among Vietnamese migrants must consider these contextual factors. Interventions should focus on fostering social inclusion, improving cultural competence among service providers, and addressing stigma related to mental health. For instance, community-based programs that encourage social engagement and accessible mental health services tailored to Vietnamese cultural norms could help alleviate loneliness and its associated psychological burdens. These measures would improve mental well-being and facilitate timely access to psychiatric care for Vietnamese migrants in Japan.

Several limitations of this study should be noted. First, the small sample size may limit the generalizability of the findings. The use of nonrandom sampling methods, including recruitment through social networking services, may have introduced selection bias, potentially leading to the underrepresentation of individuals who are less digitally connected. In addition, the cross-sectional nature of the study precludes causal inferences between loneliness and mental health outcomes. Furthermore, the demographic composition of the sample―with technical intern trainees and international students comprising approximately 40% of the participants―differs from the official residency statistics of Vietnamese migrants in Japan ^(6)^. This discrepancy suggests that the findings may not fully represent other significant subgroups, such as permanent residents or those in different employment sectors. Despite these limitations, this study provides valuable insights into the experiences of loneliness among Vietnamese migrants in Japan. By identifying the unique challenges faced by this population, the findings serve as a foundation for developing targeted interventions to improve their mental health and well-being. Future research should adopt longitudinal designs to further explore the causal relationships between loneliness and mental health outcomes, while considering the diverse subgroups within the Vietnamese migrant population.

In conclusion, this study highlights loneliness as a key factor contributing to depressive and anxiety symptoms among Vietnamese migrants in Japan. Addressing loneliness is essential to improving their mental health as the migrant population grows rapidly. Socioeconomic difficulties and limited social support exacerbate their challenges, underscoring the need for targeted interventions. Policies should focus on preventing mental health issues among Vietnamese migrants, improving access to psychiatric care, and specifically addressing loneliness as a critical factor. Future research should incorporate qualitative studies alongside other approaches to better capture the lived experiences and unique challenges faced by Vietnamese migrants in Japan, thereby informing effective, context-specific interventions.

## Article Information

### Conflicts of Interest

None

### Sources of Funding

This work was supported by JSPS KAKENHI (Grant Numbers: JP19K11277 and JP22H03420). The funder did not take part in the conceptualization, design, data collection, analysis, decision to publish, or preparation of the manuscript.

### Acknowledgement

We express our profound gratitude to all participants of the survey. We also acknowledge the substantial contributions of Saori Iwamoto from Kobe City College of Nursing and Kyoko Shimazawa from Otemae University in the design and initiation of this study.

### Author Contributions

Tadashi Yamashita ensured the integrity and accuracy of the data analysis and had full access to all study data. Tadashi Yamashita, Pham Nguyen Quy, and Kenji Kato drafted the manuscript, and all authors critically revised it for important intellectual content. Tadashi Yamashita, Kuniyasu Kamiya, and Kenji Kato performed statistical analyses and Pham Nguyen Quy, Emi Nogami, and Chika Yamada provided supervision. All authors contributed to the study’s concept and design, data acquisition, analysis, and interpretation.

### Approval by Institutional Review Board (IRB)

The study protocol was drafted in accordance with the Declaration of Helsinki and received approval from the Kobe City College of Nursing Research Ethics Review Committee (Approval No.: 20124-05). Instructions for obtaining informed consent were provided in Vietnamese. Informed consent was secured from all participants included in the study.
